# Mitotic and apoptotic activity in colorectal neoplasia

**DOI:** 10.1186/s12876-018-0786-y

**Published:** 2018-05-18

**Authors:** Darina Kohoutova, Jaroslav Pejchal, Jan Bures

**Affiliations:** 1 Charles University, Faculty of Medicine in Hradec Kralove, University Hospital Hradec Kralove, 2nd Department of Internal Medicine - Gastroenterology, Sokolska 581, 500 05 Hradec Kralove, Czech Republic; 2 University of Defence, Faculty of Military Health Sciences, Hradec Kralove, Czech Republic

**Keywords:** Mitosis, Apoptosis, Colorectal adenoma, Colorectal carcinoma

## Abstract

**Background:**

Colorectal cancer (CRC) is third most commonly diagnosed cancer worldwide. The aim of the prospective study was to evaluate mitosis and apoptosis of epithelial cells at each stage of colorectal neoplasia.

**Methods:**

A total of 61 persons were enrolled into the study: 18 patients with non-advanced colorectal adenoma (non-a-A), 13 patients with advanced colorectal adenoma (a-A), 13 patients with CRC and 17 controls: individuals with normal findings on colonoscopy. Biopsy samples were taken from pathology (patients) and healthy mucosa (patients and healthy controls). Samples were formalin-fixed paraffin-embedded and stained with haematoxylin-eosin. Mitotic and apoptotic activity were evaluated in lower and upper part of the crypts and in the superficial compartment. Apoptotic activity was also assessed using detection of activated caspase-3.

**Results:**

In controls, mitotic activity was present in lower part of crypts, accompanied with low apoptotic activity. Mitotic and apoptotic activity decreased (to almost zero) in upper part of crypts. In superficial compartment, increase in apoptotic activity was observed. Transformation of healthy mucosa into non-a-A was associated with significant increase of mitotic activity in lower and upper part of the crypts and with significant increase of apoptotic activity in all three compartments; p < 0.05. Transformation of non-a-A into a-A did not lead to any further significant increase in apoptotic activity, but was related to significant increase in mitotic activity in upper part of crypts and superficial compartment. A significant decrease in apoptotic activity was detected in all three comparments of CRC samples compared to a-A; p < 0.05. No differences in mitotic and apoptotic activity between biopsies in healthy controls and biopsy samples from healthy mucosa in patients with colorectal neoplasia were observed. Detection of activated caspase-3 confirmed the above findings in apoptotic activity.

**Conclusions:**

Significant dysregulation of mitosis and apoptosis during the progression of colorectal neoplasia, corresponding with histology, was confirmed. In patients with sporadic colorectal neoplasia, healthy mucosa does not display different mitotic and apoptotic activity compared to mucosa in healthy controls and therefore adequate endoscopic/surgical removal of colorectal neoplasia is sufficient.

## Background

Colorectal cancer (CRC) is the third most commonly diagnosed cancer with incidence of 1.36 million and mortality of 694,000 cases worldwide [1]. The etiopathogenesis of CRC is multifactorial, nevertheless, dysregulation of mitosis and apoptosis contribute to the pathogenesis of CRC significantly. Abnormalities in apoptosis are also responsible for poor response or resistance of CRC cells to chemotherapeutic agents and radiotherapy [2, 3].

The colorectal mucosa is formed into the crypts of Lieberkühn, which increase in number between the birth and adulthood [4]. In each colonic crypt there are four to six stem cells at the bottom producing the enormous number of colonocytes, which move from the lower to the upper part of crypts and reach the colonic lumen. At the lumen, they are eliminated by apoptosis [5–7]. The intestinal epithelium is fully replaced within 3–5 days [8] and the total proliferation rate is 3–10 billion colonocytes per day [5, 9]. Therefore, cell production, differentiation and apoptosis have to be regulated precisely [8].

Colorectal carcinomas arise in 70–80% sporadically [10], usually through the “non-advanced adenoma - advanced adenoma - carcinoma” sequence. Advanced colorectal adenoma is defined as a neoplasia larger than 10 mm and/or containing villous component and/or containing high grade dysplasia [11].

The aim of our prospective study was to evaluate the degree of mitotic and apoptotic activity at each stage of colorectal neoplasia.

## Methods

### Patients

A total of 18 patients with non-advanced colorectal adenoma (13 men, 5 women, aged 41–84, mean 66 ± 12; 8/18 right sided neoplasia), 13 patients with advanced colorectal adenoma (10 men, 3 women, aged 58–79, mean 67 ± 7; 4/13 right sided neoplasia) and 13 patients with colorectal carcinoma (5 men, 8 women, aged 50–87, mean 66 ± 10; CRC: 4/13 right sided neoplasia) were included into the prospective study. Inclusion criteria for those with colorectal neoplasia were: sporadic neoplasia, absence of previous colorectal surgery, negative history of inflammatory bowel disease, absence of any type of colitis (including inflammatory bowel disease, pseudomembranous colitis, ischaemic colitis), absence of serrated lesion, absence of hyperplastic polyps proximal to the rectosigmoid colon, absence of diverticulitis and postradiation proctopathy. Two patients with well differentiated carcinoma and 11 patients with moderately differentiated carcinoma were included. A total of 7 patients with dedifferentiated carcinoma were excluded from the original group of 20 patients with CRC as mitotic and apoptotic activity could not be evaluated due to presence of excessive necrotic tissue. A total of 17 controls, individuals with normal findings on colonoscopy, with no previous history of colorectal neoplasia or inflammatory bowel disease (6 men, 11 women, aged 25–75, mean 55 ± 14), were enrolled into the study.

### Collection of samples

Biopsy samples were taken during diagnostic and/or therapeutic colonoscopy. Paired biopsies were obtained in patients with colorectal neoplasia: one sample was taken from the pathology and the second sample was taken from a healthy mucosa, 10 cm distally from the side of neoplasia. In healthy individuals, one biopsy sample was obtained, usually in the rectum, after the exclusion of a colorectal neoplasia in the colon and the rectum.

All biopsy samples were formalin-fixed (10% formalin), embedded into paraffin (Paramix, Holice, the Czech Republic), and tissue sections 5 μm thick were cut (Microtome model SM2000 R, Leica, Heidelberg, Germany). Sections were stained with haematoxylin-eosin (Merck, Kenilworth, NJ, USA) and evaluated using a BX-51 microscope (Olympus Czech Group, Prague, Czech Republic). The grade of mitosis, conveyed as a mitotic index [mitotic index (%) = number of mitotic figures × 100 / number of all evaluated cells] and grade of apoptosis, conveyed as an apoptotic index [apoptotic index (%) = number of apoptotic cells × 100 / number of all evaluated cells] were assessed in three different compartments: in the superficial compartment (which is in the direct contact with the lumen of the large intestine), in the upper part of intestinal crypts and in the lower part of intestinal crypts. Single apoptotic fragments of similar size, when compared to the adjacent non-apoptotic cells, or a cluster of at least three small apoptotic bodies were considered to be apoptotic cells. A minimal amount of 750 cells were evaluated in each compartment in each biopsy sample.

**Fig. 1:**
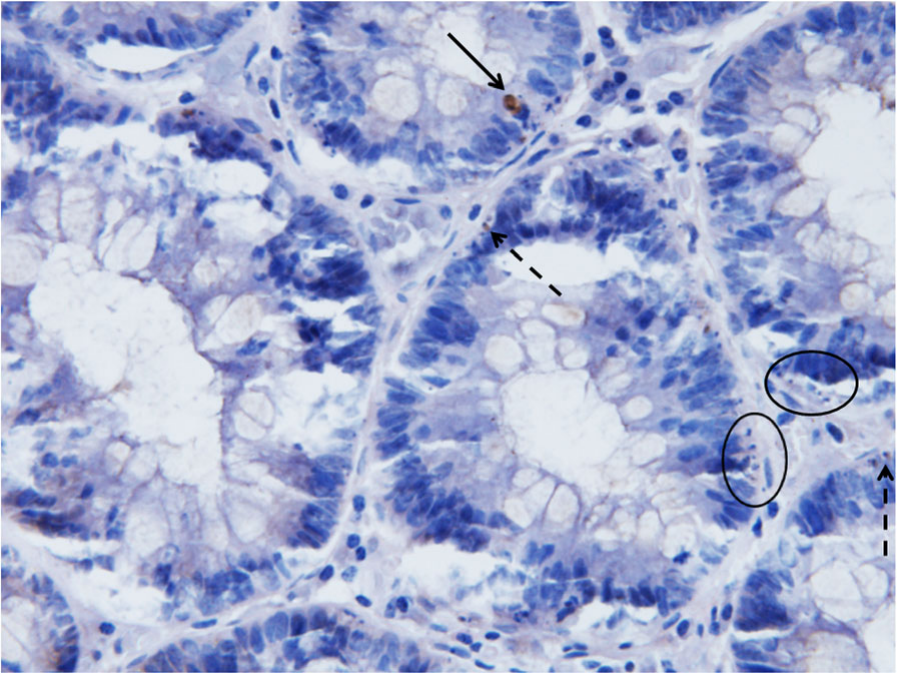
Immunohistochemical detection of activated caspase-3 counterstained with haematoxylin. Original magnification: × 500. Apoptotic cell with sufficient cytoplasmatic material and strong activated caspase-3 positivity (arrow); small apoptotic body with with weak activated caspase-3 positivity (dashed arrow); apoptotic bodies without cytoplasmatic material that would have been positive in samples stained with haematoxylin-eosin, but are not dysplaying any positivity in activated caspase-3 immuno-stained samples (circled)

Apoptotic activity was also assessed immunohistochemically by detection of activated (cleft) caspase-3, which is a key element regulating the apoptotic process. For that, a rabbit monoclonal antibody (1:200; Cell Signaling Technology, Danvers, MA, USA) and standard peroxidase technique in 5 μm thick tissue sections were used [12]. After the detection of activated caspase-3, samples were counterstained with haematoxylin (Fig. 1). Clusters of ≥3 apoptotic bodies would have been positive in haematoxylin-eosin staining, but might not have shown activated caspase-3 positivity, as apoptotic bodies were seen if the cytoplasmatic tissue was maintained. If the apoptotic body contained nuclear material only, positivity was not observed.

**Fig. 2:**
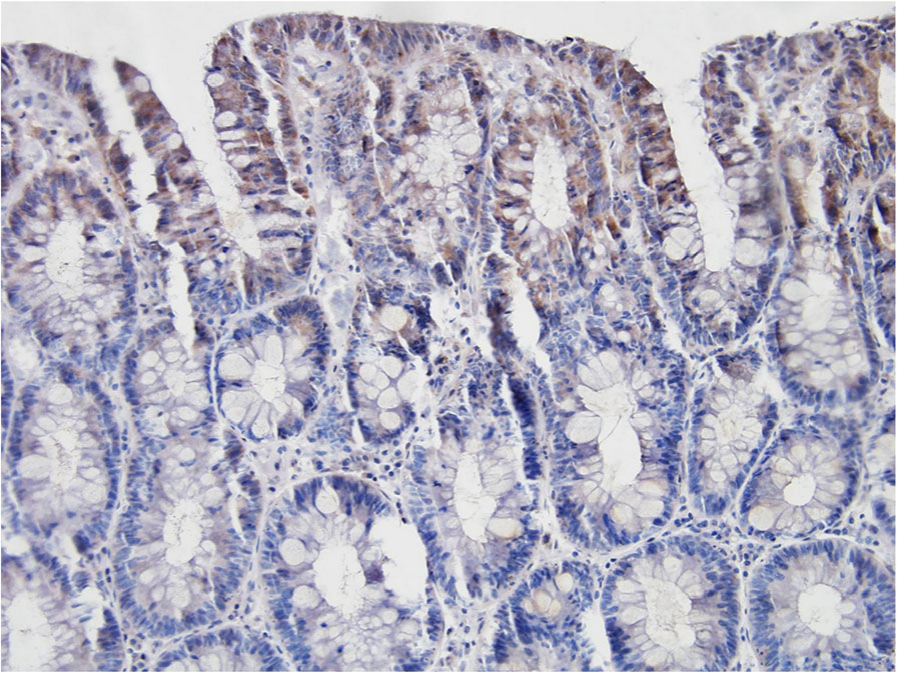
Falsely positive immunohistochemical detection of activated caspase-3 in the superficial compartment and at the outfall of crypts. The sample was counterstained with haematoxylin. Original magnification: × 200

The original effort was to evaluate apoptosis similarly to the samples stained with haematoxylin-eosin. Nevertheless, high false positivity in marginal areas was observed (Fig. 2), probably due to trauma when biopsy sample was taken. Mild intensity of trauma does not seem to induce necrotic cell death, however, it seems to be sufficient enough to result in a rapid activation of apoptotic signaling pathways. Subsequently, some samples (especially of small size) could not be assessed at all. In the remaining samples, apoptotic activity could not have been evaluated in the superficial compartment and at the outfall of the crypts. At least 1500 cells per compartment in each biopsy sample were analysed. Therefore, after an exclusion of false positive areas, apoptotic activity was evaluated in the upper and lower part of crypts. Firm conclusion were drawn from the upper cryptal compartment as sufficient numbers for statistics were obtained from this compartment only.

Preliminary data without controls in each group of patients with colorectal neoplasia and without any data on mitosis have been published previously [13].

### Statistical analysis

Obtained data were tested statistically by means of descriptive statistics and Mann-Whitney rank sum test using Statistica software, version 13, 2013, Tulsa, OK, USA.

### Ethical issues

All patients included in the study were given the necessary information and provided informed consent via a signed form. The project was approved by the Joint Ethical Committee (Charles University, Faculty of Medicine in Hradec Kralove, University Hospital Hradec Kralove); approval number: 201107 S54. For all obtained data, all personal identification information was removed in compliance with the Czech laws for protection of confidentiality.

## Results

**Fig. 3:**
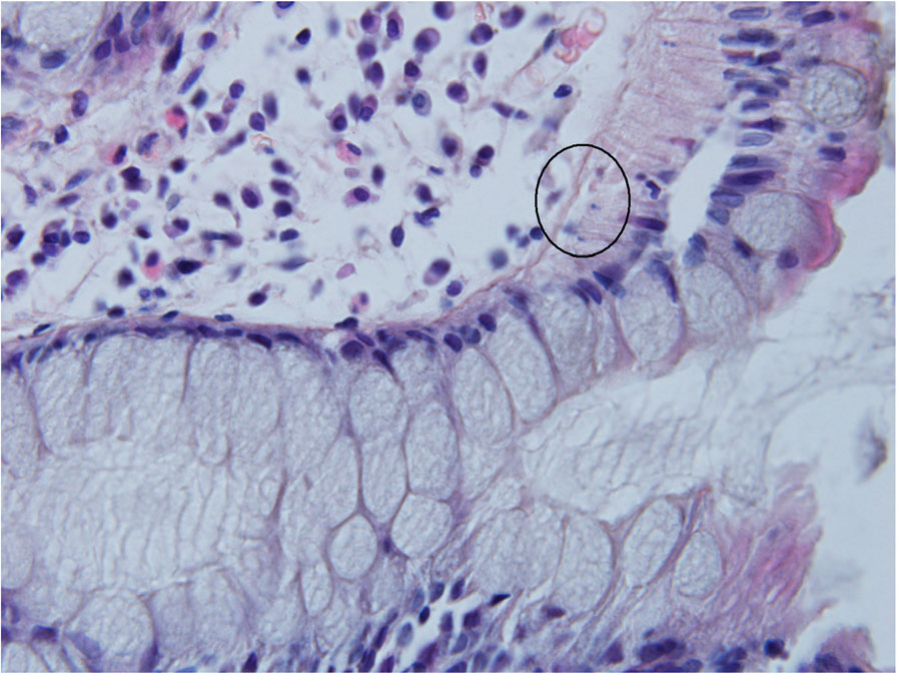
Control sample showing the upper part of the crypt with details of the cryptal orificium. No mitotic and apoptotic activity is observed in the upper part of the crypts. A cluster of apoptotic bodies is present in the superficial compartment (circled). Haematoxylin-eosin, original magification: × 600

In healthy controls, mitotic activity was observed in the lower part of crypts, where it was accompanied by low apoptotic activity. Both activities decreased (to almost zero) in the upper part of crypts (Fig. 3), whereas apoptotic activity increased again in the superficial compartment (Figs. 5, 6 and 7).

Transformation of healthy mucosa into a non-advanced adenoma (non-a-A) was associated with a significant increase of apoptotic activity in all three compartments (Figs. 5, 6 and 7); p < 0.05. The most significant increase was present in the upper part of crypts, where it was associated with a significant increase in mitotic activity (Fig. 6); p < 0.05.

**Fig. 4:**
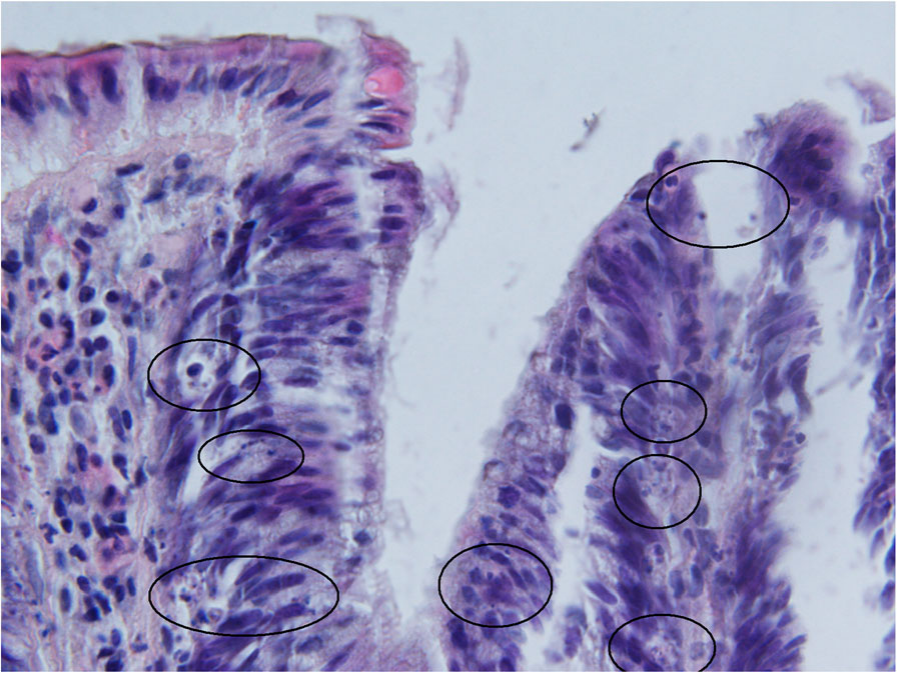
Advanced adenoma: apoptotic activity in the upper part of crypts (apoptotic bodies: circled). Haematoxylin-eosin, original magnification: × 600

**Fig. 5:**
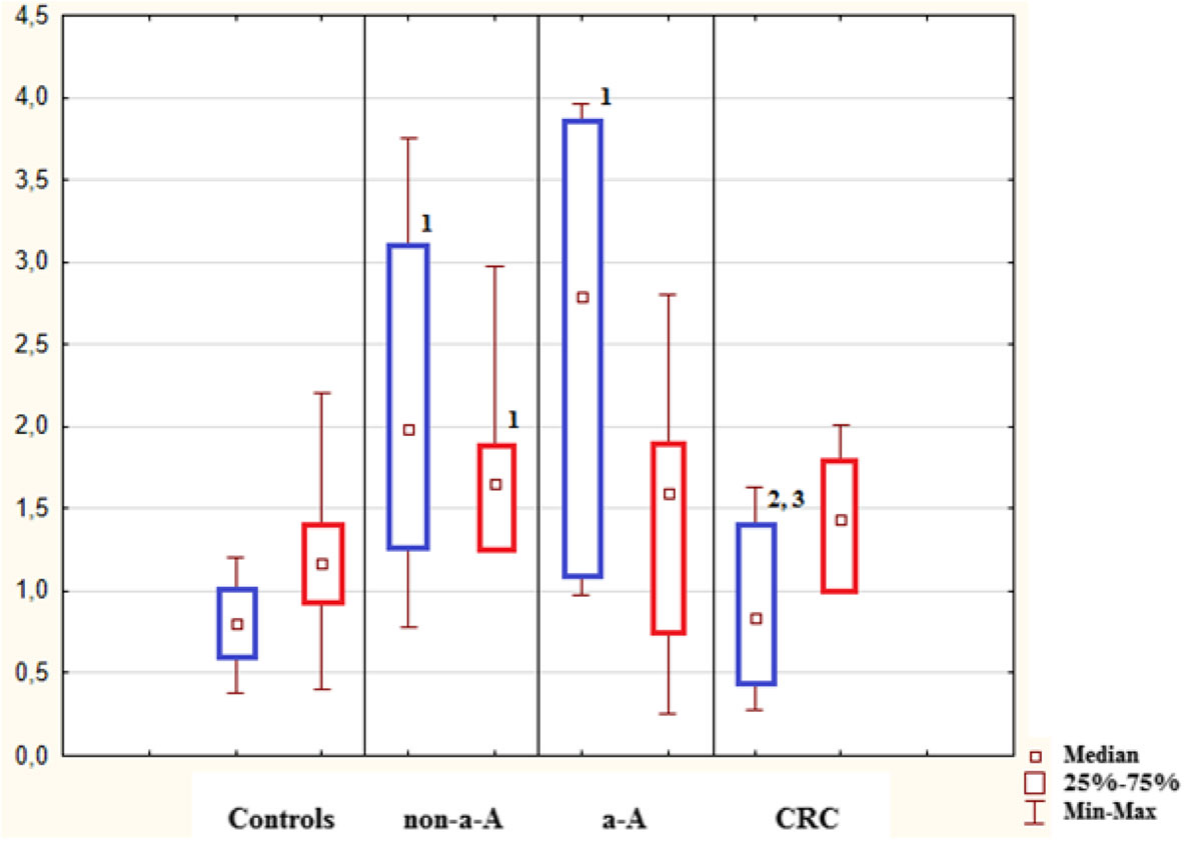
Lower part of crypts: apoptosis (in blue) conveyed as an apoptotic index (%) and mitosis (in red) conveyed as a mitotic index (%). 1: statistically significant difference when compared to control: *p* < 0.05. 2: statistically significant difference when compared to non-advanced adenoma (non-a-A): *p* < 0.05. 3: statistically significant difference when compared to advanced adenoma (a-A): *p* < 0.05

**Fig. 6:**
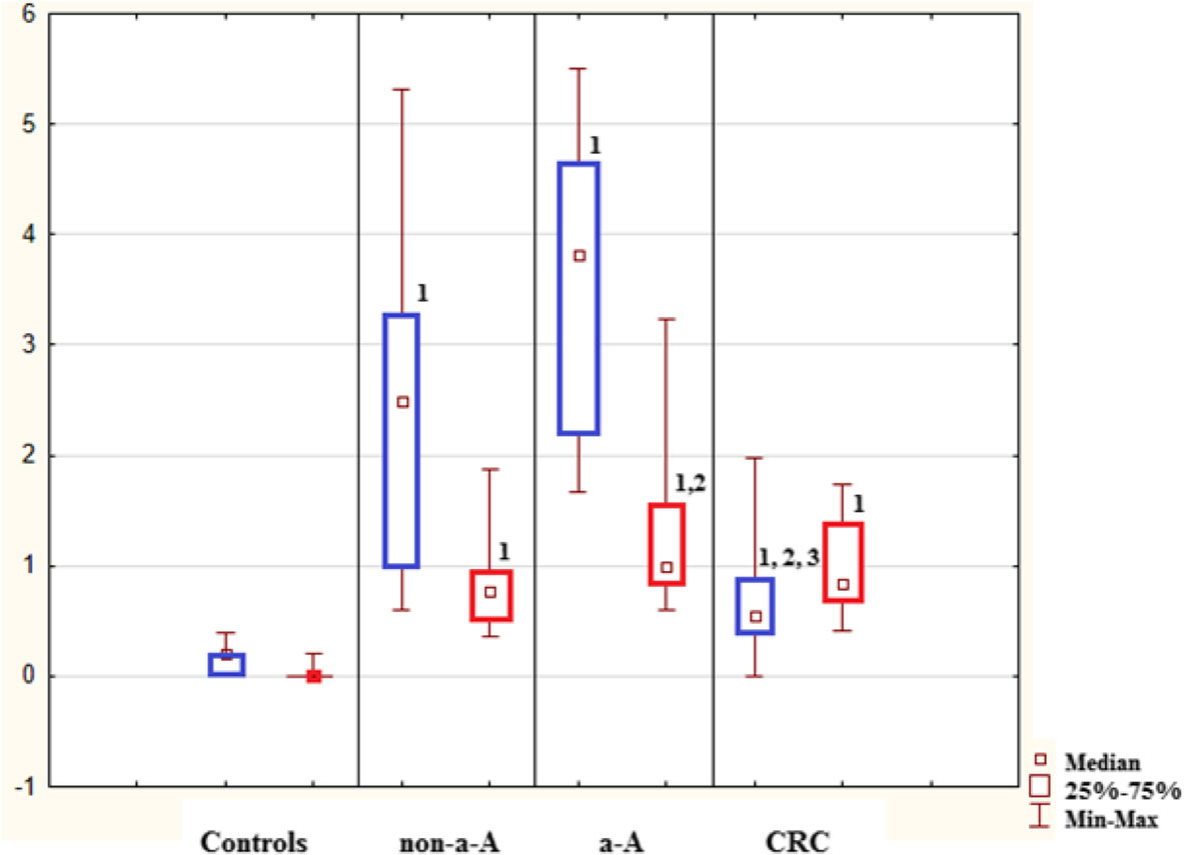
Upper part of crypts: apoptosis (in blue) conveyed as an apoptotic index (%) and mitosis (in red) conveyed as a mitotic index (%). 1: statistically significant difference when compared to control: *p* < 0.05. 2: statistically significant difference when compared to non-advanced adenoma (non-a-A): *p* < 0.05. 3: statistically significant difference when compared to advanced adenoma (a-A): *p* < 0.05

**Fig. 7:**
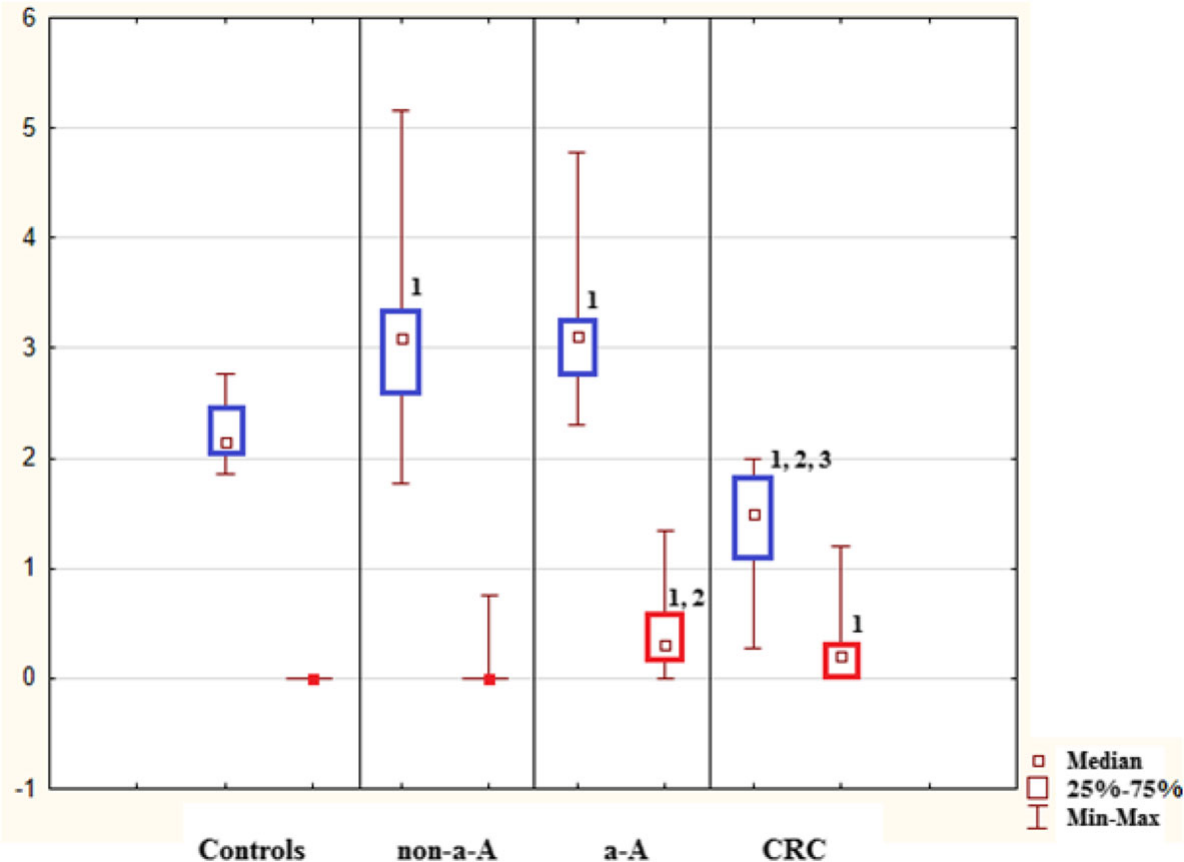
Superficial compartment: apoptosis (in blue) conveyed as an apoptotic index (%) and mitosis (in red) conveyed as a mitotic index (%). 1: statistically significant difference when compared to control: *p* < 0.05. 2: statistically significant difference when compared to non-advanced adenoma (non-a-A): *p* < 0.05. 3: statistically significant difference when compared to advanced adenoma (a-A): *p* < 0.05

Transformation of a non-a-A into an advanced adenoma (a-A) did not affect apoptotic activity (Figs. 5, 6 and 7) significantly, nevertheless, differences were found in mitotic activity: when compared to non-a-A group, significantly higher mitotic activity in the upper part of crypts and in the superficial compartment of a-A group was confirmed (Figs. 6, 7 and 4); p < 0.05.

Finally, there was a statistically significant decrease in apoptotic activity in all three compartments of carcinoma samples (CRC) compared to a-A group (Figs. 5, 6 and 7); p < 0.05.

**Fig. 8:**
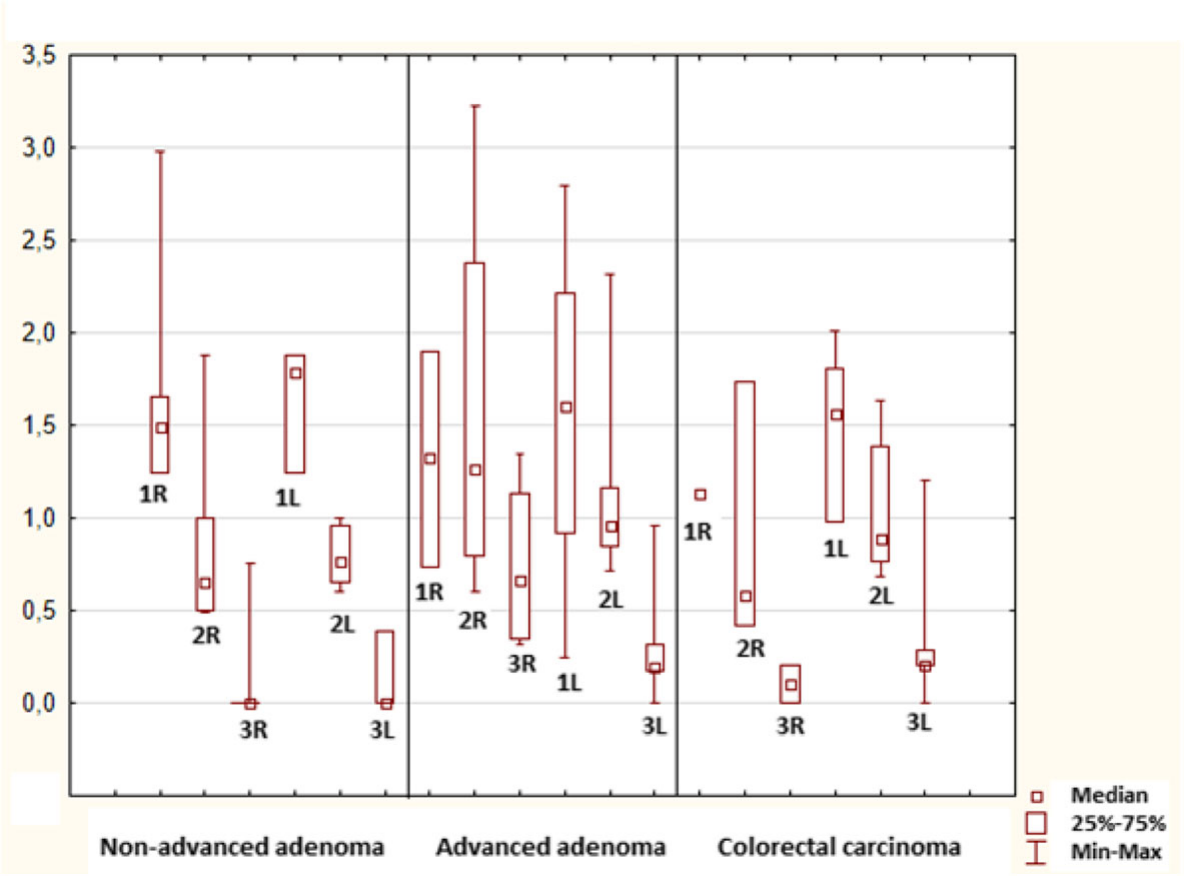
Mitotic index in the lower part of the crypts (1), upper part of the crypts (2) and superficial compartment (3) in the right and left (R, L) colon

**Fig. 9:**
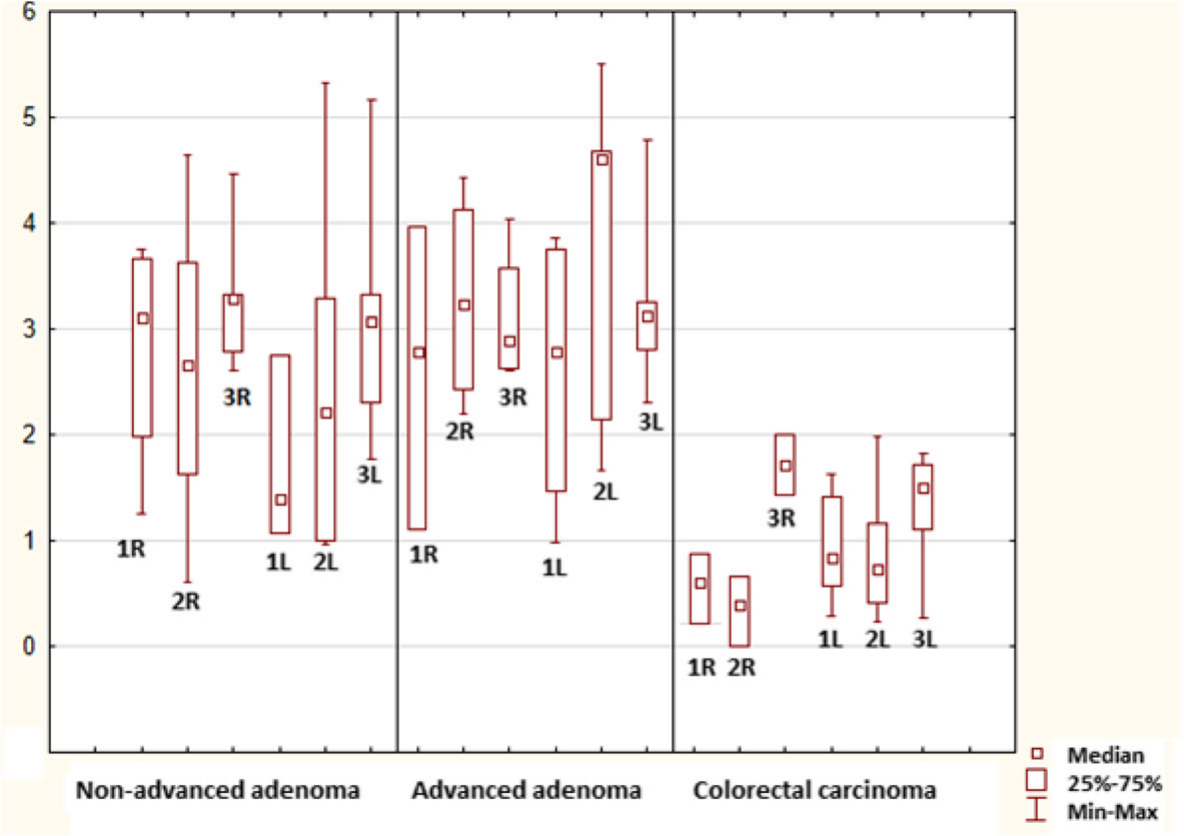
Apoptotic index in the lower part of the crypts (1), upper part of the crypts (2) and superficial compartment (3) in the right and left (R, L) colon

Further statistical analysis was conducted in order to compare mitosis and apoptosis of neoplastic epitheĺial cells between the right (caecum, ascending colon, transverse colon) and the left colon (descending colon, sigmoid colon and rectum): no differences were found in each pathology; p > 0.05, Figs. 8 and 9.

Mitotic and apoptotic activity in biopsies from the normal tissue taken in patients with colorectal neoplasia in the right and left colon was assesed: there were no differences, p > 0.05.

**Fig. 10:**
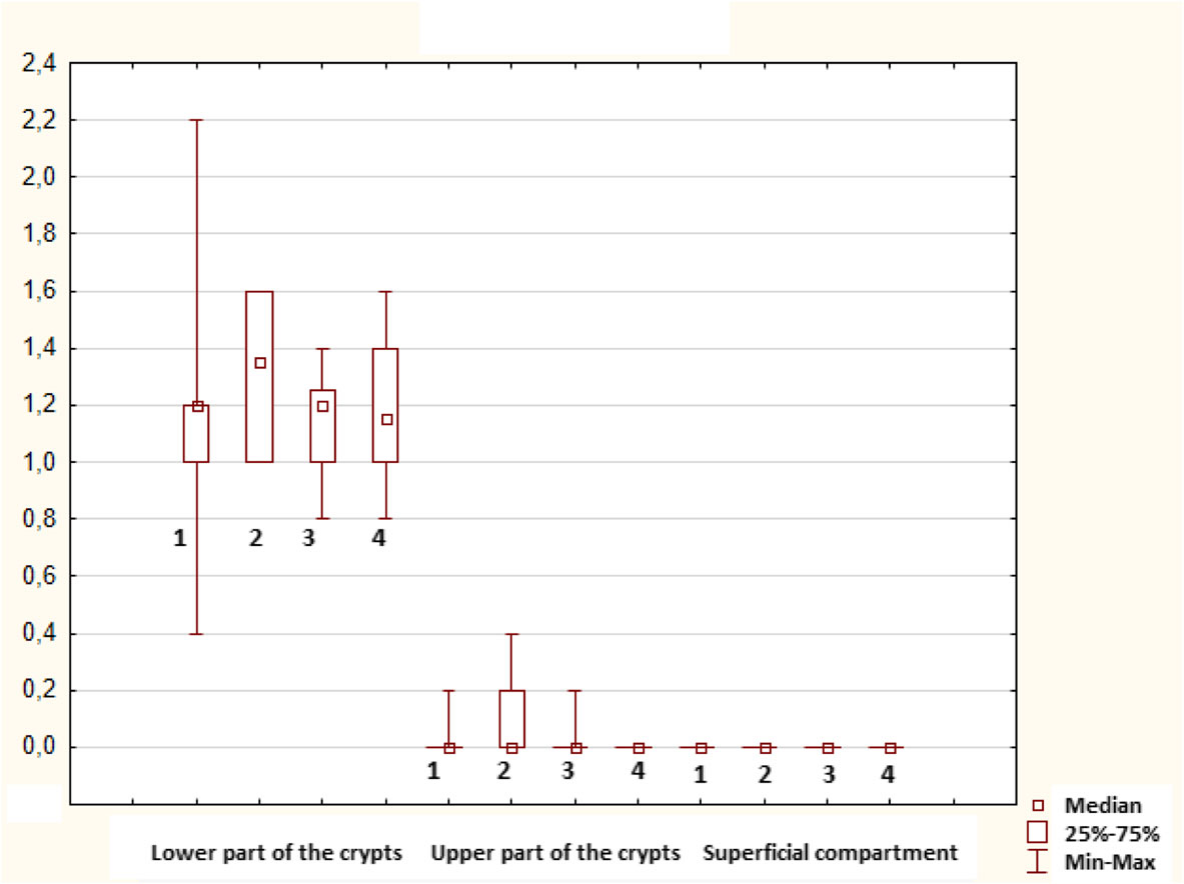
Mitotic index in controls from healthy individuals (1) and control samples in each group of patients with colorectal neoplasia (2: non-advanced adenoma; 3: advanced adenoma; 4: colorectal carcinoma)

**Fig. 11:**
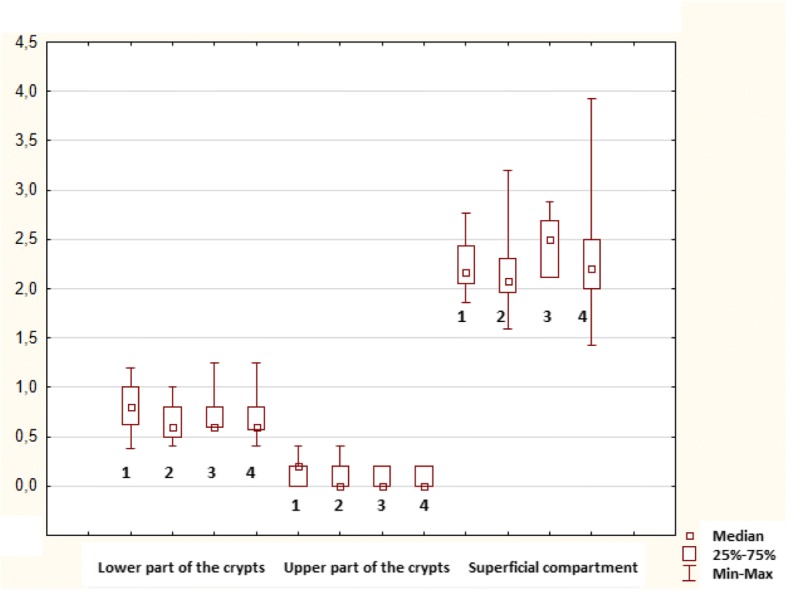
Apoptotic index in controls from healthy individuals (1) and control samples in each group of patients with colorectal neoplasia (2: non-advanced adenoma; 3: advanced adenoma; 4: colorectal carcinoma)

No differences in mitotic and apoptotic activity between controls from healthy individuals and control samples in each group of patients with colorectal neoplasia in each compartment were found; p > 0.05, Figs. 10 and 11.

A total of eight CRC patients had the neoplasia localised in the rectosigmoid junction or in the rectum and the biopsy from the healthy mucosa was taken in the rectum. Mitotic and apoptotic activity from these samples were compared with mitotic and apoptotic activity of eight randomly chosen controls, whose biopsies were taken in the rectum: no differences were found out; p > 0.05.

No differences in mitotic and apoptotic activity between females and males were observed; p > 0.05.

Immunohistochemical detection of activated caspase-3 showed significant increase in apoptotic activity in the upper part of the crypts during the transformation of healthy mucosa into a non-a-A and an a-A; p < 0.05. This was followed by a significant decrease of activated caspase-3 positivity in the same compartment in CRC samples when compared to a non-a-A and an a-A; p < 0.05 (Table 1).

**Table 1:** Immunohistochemical detection of activated caspase-3 positive cells in the upper part of crypts

	Apoptotic index of activated caspase-3 positive cells in the upper part of crypts (%)
Controls	Median 0.00, IQR 0 (13)
Non-advanced-adenoma	Median 1.37, IQR 1.42–1.18 (8) ^1^
Advanced adenoma	Median 1.84, IQR 2.03–1.72 (10) ^1, 2^
Colorectal carcinoma	Median 0.86, IQR 1.05–0.77 (9) ^1, 2, 3^

Results are conveyed as median and interquartile range (IQR) of apoptotic indices. Number of compartments evaluated in particular group are in brackets

1: statistically significant difference when compared to control: p< 0.05

2: statistically significant difference when compared to non-advanced adenoma (non-a-A): p< 0.05

3: statistically significant difference when compared to advanced adenoma (a-A): p< 0.05

## Discussion

Dysregulation of epithelial proliferation and apoptosis is typical for a neoplastic process. Recently, therapeutic strategies involving induction of apoptosis have become an effective part of therapy of an established carcinoma [14–18], but it is not yet a tool to prevent development of carcinoma from precancerous lesions.

The “programmed cell death” was first proposed by Lockshin and Williams in 1965 [19]. The term “apoptosis” was first introduced by Kerr et al. in 1972, who suggested, that this process involves a controlled cell deletion, which plays a complementary, but opposite role to mitosis [20].

In healthy mucosa, mitotic activity was observed in the lower part of crypts only, where it was accompanied by a certain level of apoptotic activity. Apoptotic activity in this part of crypts might be a mechanism contributing to the maintenance of genetic information providing stability of mutiplying stem cells and progenitors [21, 22]. The upper part of a crypt is a compartment where the cells undergo differentiation [23] and mitotic and apoptotic activity decrease to almost zero in healthy controls. Apoptotic activity increases in the superficial compartment again, where the cells are eliminated physiologically. Our study confirmed these findings in all control samples taken from both healthy individuals and patients with colorectal neoplasia.

In this study, mitosis and apoptosis increased significantly almost in all evaluated compartmens of non-advanced and advanced adenomas. Resembling trends in both neoplasias suggest that both these processes are triggered by genetic alterations. On the contrary, mitotic activity remained unaffected at the luminal surface of non-advanced adenomas indicating maintenance of terminal differentiation in this compartment despite altered morphology, which is in agreement with the literature [24]. This was not observed in advanced adenomas. The transformation of a non-advanced adenoma into an advanced form was associated with increased mitotic activity in the upper cryptal and superficial compartment, which implies further distortion of mucosal spatial differentiation. The final step of adenoma – carcinoma sequence was related to decrease in apoptosis in all compartments, whereas increased mitotic activity remained unchanged. Amplified production of “incompetent” cells, which are neither eliminated sufficiently, contribute to further progression of growth and development of invasive colorectal carcinoma. In our study, activation of caspase-3 correlated with morphological findings in the upper part of crypts, i.e. the comparment with the most profound dysregulation of mitotic and apoptotic activity in all selected colonic neoplasms.

Apoptosis is regulated by two major mechanisms: Bcl-2 family, which consists of pro- and antiapoptotic members and Inhibitor of apoptosis (IAP) family [25]. Survivin is the smallest member of the IAP family of proteins and is involved in inhibition of apoptosis, regulation of cell cycle and resistence to chemotherapy. Survivin is highly expressed during embryonal period, absent in majority of differentiated tissues, but upregulated in different human cancers [26]. Soreide et al. documented that survivin is an independent predictor for metachronous colorectal carcinoma development in patients with a sporadic colorectal adenoma [27]. Hernandez et al. looked at expression of survivin in colorectal adenomas and carcinomas and confirmed not only overexpression of survivin in these tissue samples, but interestingly, increase in expression of survivin during the normal mucosa-adenoma-carcinoma sequence [28]. Konturek et al. reported that a 14-day therapy with celecoxib, inhibitor of cyclooxygenase-2, lead to a significant decrease of survivin in tumour tissue and normal rectal mucosa of patients with rectal adenocarcinoma [29]. Survivin therefore seems to be an ideal tumour marker and potential therapeutic target [30, 31].

Mutations in the WNT/beta-catenin pathway are typical for sporadic colorectal cancer and apoptosis in CRC cells with these mutations is induced by histone deacetylase inhibitors [32]. Butyrate, a short-chain fatty acid which is produced from dietary fiber by intestinal microbiota, is the most well-known inhibitor of histone deacetylase [33, 34]. Butyrate is the preferred source of energy for healthy colonocytes [35] nevertheless, due to its different concentration in each compartment, the effect of butyrate is diverse: low concentrations of butyrate, which are typical for the base of colonic crypts, are quickly metabolized and stimulate cell proliferation. Higher concentrations of butyrate, which are present near the intestinal lumen, exceed the metabolic capacity of colonocytes. This results in accumulation of butyrate in the nucleus where it acts as a histone deacetylase inhibotor and induces apoptosis [36]. Our study, when contemplating the degree of mitosis and apoptosis in the lower part of crypts and in the superficial compartment, fulfills these theoretical prerequisites derived from the effect of butyrate.

Due to Warburg effect, the cells of colorectal neoplasia prefer glucose as the energy source. Butyrate therefore accumulates in the nuclei of cancerous colonocytes and induces apoptosis via the inhibition of histone deacetylase [36]. Our study showed that apoptosis increased significantly in all compartments in non-advanced and advanced adenomas, which is in agreement with a study conducted by Strater et al. [7]. Nevertheless, significant decrease in apoptosis was observed during the transformation of advanced colorectal adenomas into carcinomas. This finding is in agreement with a study performed by Kikuchi et al. [37], but the assumptions derived from Warburg effect are not fulfilled. This could be explained by the fact, that adenoma cells are known to be very sensitive to the effects of butyrate on apoptosis and proliferation suppression, more when compared to carcinoma cells (32). Further, there are other components regulating apoptosis, such as antiapoptotic survivin. Pathways induced by mitogens secreted from apoptotic cells, such as AKT, JAK/STAT and ERK, play a key role in the complex process of apoptosis, too [28, 32, 38, 39].

No differences in mitotic and apoptotic activity were found between right sided and left sided neoplasias, between normal tissue obtained in patients with right sided and left sided neoplasias, and between males and females. Still, we are very carefull to draw any firm conclusions - despite a very high number of cells (> 750) were evaluated in each compartment - as the numbers of participants in each subgroup (especially those with a right sided advanced adenoma and CRC (4/13) and women in the group with advanced adenoma (3/13)) were small.

Another limitation worth noting is that most of the samples taken from healthy patients were from the rectum and we are aware that rectal samples do not have to be fully representative of the entire colorectum. Yet, based on our study, the observation of no difference in mitotic and apoptotic activity between controls from healthy individuals and control samples in each group of patients with colorectal neoplasia is of a very high clinical significance: adequate endoscopic/surgical removal of colorectal neoplasia is warranted, but should be sufficient in case that we deal with a sporadic colorectal neoplasia in a patient without inflammatory bowel disease.

## Conclusions

Significant dysregulation of mitosis and apoptosis during the progression of colorectal neoplasia, corresponding with histology, was confirmed. In patients with sporadic colorectal neoplasia, healthy mucosa does not display different mitotic and apoptotic activity compared to mucosa in healthy controls and therefore adequate endoscopic/surgical removal of colorectal neoplasia is sufficient.

## Abbreviations

a-A: Advanced colorectal adenoma
CRC: Colorectal carcinoma
non-a-A: Non-advanced colorectal adenoma
